# Blocking of FcR Suppresses the Intestinal Invasion of Scrapie Agents

**DOI:** 10.1371/journal.pone.0017928

**Published:** 2011-03-18

**Authors:** Ryuta Uraki, Akikazu Sakudo, Kosuke Michibata, Yasuhisa Ano, Jyuri Kono, Masayoshi Yukawa, Takashi Onodera

**Affiliations:** 1 Department of Molecular Immunology, School of Agricultural and Life Sciences, University of Tokyo, Tokyo, Japan; 2 Laboratory of Biometabolic Chemistry, School of Health Sciences, Faculty of Medicine, University of the Ryukyus, Nishihara, Okinawa, Japan; 3 Department of Veterinary Medicine, College of Bioresource Sciences, Nihon University, Fujisawa, Kanagawa, Japan; Creighton University, United States of America

## Abstract

Prion diseases are a family of neurodegenerative zoonotic foodborne disorders. Although prions can be transmitted orally, the mechanism by which prions are incorporated into the intestine remains unclear. Our previous studies have shown that an abnormal isoform of prion protein (PrP^Sc^), which is the main component of prions, was efficiently incorporated into the intestine in suckling mice but not in weaned mice. Furthermore, suckling SCID mice lacking maternal antibodies showed decreased uptake of PrP^Sc^ into the intestine compared with suckling wild-type mice, while the lack of PrP^Sc^ uptake into the intestine of suckling SCID mice was rescued by the oral administration of IgG. These findings raise the possibility that the neonatal Fc receptor (nFcR), which contributes to the uptake of maternal antibodies into the intestine, plays a role in PrP^Sc^ incorporation into the intestine. The present immunohistochemical study further showed that the FcR blocker Z-ε-aminocaproic acid (ZAA) inhibited PrP^Sc^ incorporation into the intestinal villi of suckling mice, supporting the above mentioned concept. Therefore, our findings provide strong evidence that nFcR and maternal antibodies are involved in PrP^Sc^ incorporation into the intestine before the weaning period.

## Introduction

Prion diseases are a unique category of illness, the pathogenesis of which is related to conformational changes in the normal protein, PrP^C^ (cellular prion protein), to a form with a high β-sheet content, PrP^Sc^ (abnormal prion protein), that is protease resistant and infectious [1], [2]. These diseases include bovine spongiform encephalopathy (BSE) in cattle, scrapie in sheep, and Creutzfeldt-Jakob disease (CJD) in humans. The appearance of variant CJD (vCJD) has raised public health concerns that BSE might be transmissible to humans across species through dietary exposure to BSE-contaminated foodstuffs [3]. In addition, human cases of vCJD have recently emerged in the UK, many years after the eradication of BSE from the country, due to the very long incubation times of prion diseases, which range from months to decades [2].

Epithelial M cells are considered to be involved in the transmigration of PrP^Sc^ from the gut and into the lymphoid system during oral infection [4]. Results from studies using artificial M cells have also indicated a role for M cells in prion absorption [5]. On the other hand, PrP^Sc^ was detected by immunohistochemistry in villous lacteals and the submucosal lymphatic system from 15 min to 3.5 h post-challenge and also in dendritic-like cells in the draining lymph nodes until 24 h post-challenge. This suggested a transepithelial pathway for prion entry through the mucosal epithelium rather than a pathway through M cells in Peyer's patches [6]. Therefore, two processes have been hypothesized to account for intestinal prion entry, the M cell dependent pathway and the M cell independent pathway. In the former route, PrP^Sc^ passes through dendritic cells and accumulates in mesenteric lymph nodes, prior to invading neurons. On the other hand, in the M cell independent pathway, PrP^Sc^ is taken up by epithelial cell transport and directly accumulates in the enteric nervous system (ENS). The former is the most accepted pathway, whereas the latter was only suggested recently [6], [7]. Furthermore, it has been reported that during the suckling and weaning periods, when Peyer's patches have not developed sufficiently, some PrP^Sc^ was detected in the dome epithelium but most was incorporated through the villous epithelia of Peyer's patches. This indicated that uptake through the villi is important for the intestinal epithelial invasion of PrP^Sc^ [8]. In addition, the levels of PrP^Sc^ incorporated by suckling SCID mice lacking maternal immunoglobulins (Ig) [9] were significantly lower than those taken up by wild-type suckling mice. Interestingly, the amount of PrP^Sc^ incorporated by suckling SCID mice was increased when immunoglobulin G (IgG) was administered orally together with PrP^Sc^. It was therefore suggested that maternal immunoglobulins or the neonatal Fc receptor (nFcR), which is expressed on columnar epithelial cells and is responsible for taking up maternal antibodies into the body, play a role in the incorporation of PrP^Sc^ through epithelial cells [8]. However, there is no evidence for a relationship among PrP^Sc^ and IgG.

**Figure 1:**
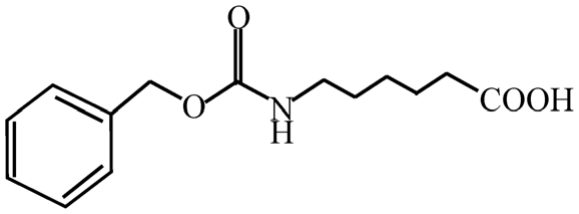
Structure of Z-ε-aminocaproic acid (ZAA). Z-ε-aminocaproic acid is a derivative form of ε-aminocaproic acid which is an analogue of the amino acid lysine.

In the present study, in order to elucidate the role of FcR in PrP^Sc^ incorporation, the effect of the FcR blocker Z-ε-aminocaproic acid (ZAA) (Fig. 1) [10] on PrP^Sc^ incorporation was analyzed.

## Results

### Incorporation of IgG through the Villi is Suppressed by ZAA in CD-1 and SCID Mice

**Figure 2:**
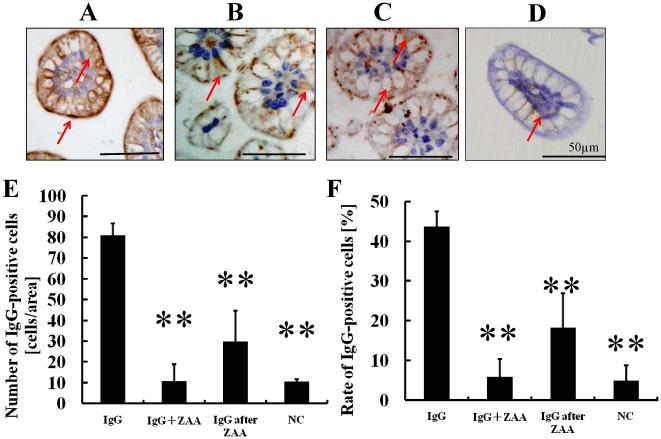
Incorporation of IgG through the villi. Histochemical analysis of IgG in the intestinal villi of 15-day-old CD-1 mice that had been orally administered IgG (A: IgG), IgG after ZAA treatment (B: IgG after ZAA), or IgG and ZAA at the same time (C: IgG + ZAA). IgG was readily incorporated into the villi in A and partially incorporated in B and C. The number and percentage of ileal epithelial cells incorporating IgG were significantly higher in A than B and C. As a negative control, IgG was stained without exogenous IgG as D. The number of IgG-positive cells (E) and the ratio of IgG-positive cells (F) are expressed as the mean ± SD. The statistical significance of differences compared to oral administration of IgG was determined using One-way analysis of variance followed with Tukey's Multiple Comparison Test (Prism 4.03, GraphPad Software, Inc., La Jolla, CA, USA). **p<0.01.

Immunohistochemistry was applied to detect IgG using sheep anti-mouse IgG. IgG was detected in the villi in the group in which only IgG was administered (Fig. 2). On the other hand, the incorporation of IgG was significantly decreased in the group in which IgG was administered after Z-ε-aminocaproic acid treatment as well as in the group in which IgG and ZAA were administered at the same time (Fig. 2). Supporting this observation, number of IgG-positive cells (cells per area) and ratio of IgG-positive cells (%) were significantly decreased in the groups of simultaneous and separate administration of ZAA with IgG compared to those of IgG administration only. Inhibitory effects of 86.9% and 59.5%, respectively, were detected in the latter two groups (Table 1). This suggested that ZAA suppressed the incorporation of IgG due to the inhibition of FcR by ZAA.

**Table 1:** The inhibitory effect of ZAA on the incorporation of IgG and PrP^Sc^ on the basis of the ratio of IgG- or PrP^Sc^-positive cells.

	Treatment	Inhibitory effect[%]
Incorporation of IgG in CD-1 mice	Administration of IgG after ZAA treatment	86.9±8.9
	Simultaneous administration of ZAA and IgG	59.5±19
Incorporation of PrP^Sc^ in CD-1 mice	Administration of PrP^Sc^ after ZAA treatment	70.1±18.7
	Simultaneous administration of ZAA and PrP^Sc^	51.5±18.5

### Incorporation of PrP^Sc^ through the Villi is Suppressed by ZAA in CD-1 Mice

**Figure 3:**
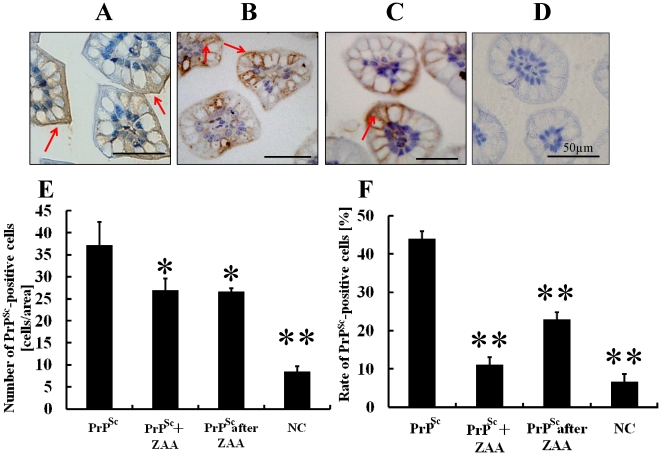
Incorporation of PrP^Sc^ through the villi in CD-1 mice. Histochemical analysis of PrP^Sc^ in the intestinal villi of 15-day-old CD-1 mice that had been orally administered PrP^Sc^ (A: PrP^Sc^), PrP^Sc^ after ZAA treatment (B: PrP^Sc^ after ZAA), or PrP^Sc^ and ZAA at the same time (C: PrP^Sc^ + ZAA). The number and percentage of ileal epithelial cells incorporating PrP^Sc^ were significantly lower in B and C than in A. D shows the staining of the PrP without exogenous PrPSc supplementation as a negative control. In our study, we could not detect the normal PrP. The number of PrP^Sc^-positive cells (E) and the ratio of PrP^Sc^-positive cells (F) are expressed as the mean ± SD. The statistical significance of differences compared to oral administration of PrP^Sc^ was determined using One-way analysis of variance followed with Tukey's Multiple Comparison Test (Prism 4.03, GraphPad Software, Inc., La Jolla, CA, USA). *p<0.05, **p<0.01.

After the effect of ZAA had been confirmed, PrP^Sc^ was detected by immunohistochemistry using the T2 antibody. PrP^Sc^ was incorporated through the villous epithelium and detected in the lamina propria in the group in which only PrP^Sc^ was administered (Fig. 3). However, the incorporation of PrP^Sc^ into the villi was significantly decreased in the group in which PrP^Sc^ was administered after ZAA treatment as well as in the group in which PrP^Sc^ and ZAA were administered at the same time (Fig. 3). Such observation was quantitatively confirmed in the terms of number of PrP^Sc^-positive cells (cells per area) and ratio of PrP^Sc^-positive cells (%). Inhibitory effects of 70.1% and 51.5%, respectively, were seen in the latter two groups (Table 1). This suggested that ZAA suppressed the incorporation of PrP^Sc^ by inhibiting FcR.

## Discussion

Although the sites at which prions invade the human body are disputed, natural infection is supposed to occur orally, especially through the gut. However, the route of prion disease invasion from the gut to the central nervous system (CNS) still remains unclear. Previous reports have shown that immune cells participate in the pathogenesis of murine prion diseases [11], [12]. In the oral transmission route, PrP^Sc^ moves through the intestinal epithelial barrier and several other biological barriers before finally reaching the CNS. To successfully invade through the intestinal epithelial barrier, pathological agents must avoid digestion in the gastrointestinal tract. Although normal proteins are digested by gastric acid in the stomach and various enzymes in the gastrointestinal tract, most PrP^Sc^ peptides are not digested because they are resistant to protease activity due to their abundant β-sheet structures. After withstanding digestion in the gastrointestinal tract and reaching the intestinal epithelial wall, the infectious agent needs to penetrate the intestinal epithelial barrier and reach the peripheral nervous system.

PrP^Sc^ was previously detected in the enteric nervous system (ENS) of an orally challenged rodent model of TSE, suggesting that PrP^Sc^ enters the body via the intestinal mucosa [13]. A number of putative receptors for prions have been reported, with one of the most interesting receptors being the 37 kDa laminin receptor precursor (LRP). LRP is incorporated into the 67 kDa mature laminin receptors (LR) expressed in the intestinal brush border of 40% of human subjects [14], [15]. The mechanism postulated for the entry of PrP^Sc^ after its ingestion involves the binding of PrP^Sc^ to the LRP followed by its internalization. It is considered that epithelial M cells might be involved in the transmigration of PrP^Sc^ from the gut and into the lymphoid system [4]. Furthermore, an experiment using artificial M cells also indicated a role for M cells in prion absorption [5]. PrP^Sc^ has also been detected in villous epithelial cells and is resistant to degradation by gastric juices and intestinal enzymes because of its abundant stable β-sheet structure.

In a previous study, the amount of PrP^Sc^ incorporated by suckling SCID mice lacking maternal immunoglobulins [9] was significantly lower than that taken up by wild-type suckling mice [8]. Moreover, the incorporation of PrP^Sc^ was upregulated when IgG was administered together with PrP^Sc^. Therefore, it was suggested that maternal immunoglobulins and nFcR play roles in the incorporation of PrP^Sc^ through epithelial cells. The nFcR binds to the IgG antibodies ingested in maternal milk and transports them through enterocytes to the systemic circulation of the newborn [16]. Furthermore, it was reported using nFcR-knockout mice that foreign proteins were complexed with immunoglobulins and incorporated via immunoglobulin endocytosis during the suckling period [17]. This shows that the Fc receptor plays a key role in PrP^Sc^ infection.

In the present study, we focused on the changes in the incorporation of PrP^Sc^ and IgG after the blocking of nFcR. As described, the amount of IgG incorporated was decreased by ZAA treatment, and the degree of PrP^Sc^ incorporation in suckling CD-1 mice administered PrP^Sc^ and ZAA was significantly decreased compared with that in suckling CD-1 mice who were administered PrP^Sc^ alone. These findings support those of our previous reports and show that nFcR is associated with the incorporation of PrP^Sc^ [1]. By contrast, experiments from Klein et al. [18] assessed the effect of Fcγ receptors on prion pathogenesis in mice deficient in Fcγ receptors I, II and III and found no effect on prion pathogenesis in these knockout mice. To address this discrepancy, further *in vivo* experiments using mice with ZAA before and after prion infection would be necessary to determine the effect of blocking Fc receptors on disease progression in their model.

## Materials and Methods

### Experimental Animals

Fifteen-day-old CD-1 mice (Japan CLEA, Tokyo, Japan) were housed in specific pathogen-free (SPF) conditions under an alternating 14 h/10 h light/dark cycle. The animals were given free access to standard laboratory food (Oriental Yeast Co., Ltd., Tokyo, Japan) and tap water and were treated in accordance with the procedures authorized by the Animal Experiment Committee of Nihon University College of Bioresource Sciences. This experiment was permitted by the guidelines for the care and use of laboratory animals approved by the College of Bioresource Science, Nihon University (permit number; NUBS-V168).

### Administration of IgG with ZAA

To confirm the effect of ZAA (patent No.: WO2004/058747), a derivative form of ε-aminocaproic acid [10], the following two experiments were performed: First, 15-day-old CD-1 and SCID mice (n = 3) were administered phosphate buffered saline (PBS) containing ZAA (30 mg/ml). Then, IgG (5 mg/ml) was administered 2 h later. The administration of IgG was repeated 3 h later, and the mice were euthanized with ether at 1 h post-administration (p.a.). In the second experiment, the same age mice were administered IgG (5 mg/ml) diluted with PBS containing ZAA (30 mg/ml). The administration of IgG was repeated 3 h later, and the mice were euthanized with ether at 1 h post-administration (p.a.).

### Administration of PrP^Sc^ with ZAA

Two experiments were performed in order to investigate whether the Fc receptor inhibitor, ZAA, affects the incorporation of PrP^Sc^. First, 15-day-old CD-1 mice (n = 3) were administered PBS containing ZAA (30 mg/ml). After 2 h, they were then administered 10 mg/ml emulsion of mouse brains infected with mouse-adapted scrapie (PrP^Sc^) (Tsukuba 1 strain [19]) diluted with PBS containing ZAA (30 mg/ml). Then, the administration of PrP^Sc^ was repeated 3 h later, and the mice were euthanized with ether at 1 h post-administration (p.a.). In the second experiment, the same age mice were administered 10 mg/ml emulsion of mouse brains infected with mouse-adapted scrapie diluted with PBS containing ZAA (30 mg/ml). Then, the administration of PrP^Sc^ was repeated 3 h later, and mice were euthanized with ether at 1 h post-administration (p.a.) the.

### Preparation of Tissue Specimens

After the mice had been euthanized with ether, their intestines were removed and fixed by immersion in PBS containing 4% paraformaldehyde for 2 h before being washed in PBS containing 6.8% sucrose. After dehydration in 100% acetone for 1 h, the tissue samples were embedded in resin (Technovit 8100; Heraeus Kulzer, Wehrheim, Germany) in accordance with the manufacturer's instructions and sectioned at a thickness of 4 µm.

### Immunohistochemistry for IgG and PrP

The resin sections obtained from the mice treated with IgG were pretreated with 0.1% CaCl_2_ at pH 7.8 containing 0.01% trypsin for 10 min at 37°C and then quenched in 0.3% hydrogen peroxide in methanol for 30 min. For IgG detection, goat anti-mouse IgG antibody (4 µg/ml; Nichirei, Tokyo, Japan) was directly incubated at room temperature for 30 min. For detection, incubation with mouse anti-PrP monoclonal antibody (T2, 10 µg/ml) was performed at 37°C for 2 h followed by incubation with goat anti-mouse IgG antibody (4 µg/ml; Nichirei, Tokyo, Japan) for 30 min. Diaminobenzidine (DAB; Wako, Osaka, Japan) was applied for 10 min, and then the sections were counterstained with hematoxylin for 1 min. The number of IgG positive cells in each microscopic visual field was counted at five random points in the villous epithelium. Cell counts are expressed as the mean ± SD of microscopic fields viewed at ×400 magnification. Intestinal epithelial cells were used to determine the intensity of infection, which was not known to the observer assessing the respective intestinal sections. And the inhibitory effect was calculated as the following formula.

The inhibitory effect  =  (percentage of ileal epithelial cells incorporating IgG orPrP^Sc^ with ZAA treatment)/(percentage of ileal epithelial cells incorporating IgG orPrP^Sc^ without ZAA treatment) ×100

The statistical significance of differences compared to oral administration of IgG or PrP^Sc^ was determined using One-way analysis of variance followed with Tukey's Multiple Comparison Test (Prism 4.03, GraphPad Software, Inc., La Jolla, CA, USA) and the inhibitory effect was analyzed by student's t-test. * *p*<0.05 **p<0.01.
